# Transcriptome profiling of peripheral blood mononuclear cells from highly susceptible adult cattle infected with a virulent strain of *Babesia bovis*

**DOI:** 10.1186/s13071-025-07126-x

**Published:** 2025-12-15

**Authors:** Janaína Capelli-Peixoto, Reginaldo G. Bastos, Anna L. McDonald, Jacob M. Laughery, Sascha Duttke, Carlos E. Suarez, Chungwon J. Chung, Massaro W. Ueti

**Affiliations:** 1 Department of Veterinary Microbiology and Pathology, College of Veterinary Medicine, Washington State University, Pullman, WA 99164-7040 USA; 2 Animal Disease Research Unit, USDA-ARS, Pullman, WA 99164-6630 USA; 3 School of Molecular Biosciences, College of Veterinary Medicine, Washington State University, Pullman, WA 99164-7520 USA

**Keywords:** Bovine babesiosis, Adult cattle, Immune cells, PBMC, RNA-Seq data

## Abstract

**Background:**

Bovine babesiosis is a tick-borne disease that poses a significant economic threat to cattle industries in tropical and subtropical areas, and *Babesia bovis* is the most virulent causative agent of bovine babesiosis. This apicomplexan parasite infects erythrocytes of cattle, causing severe hemolytic disease, and animals that survive an acute infection become persistently infected for life. Adult cattle (> 1 year of age) are highly susceptible and often succumb to acute infection. Protective host immunity involves peripheral blood mononuclear cells (PBMCs) including monocytes, dendritic cells (DC), natural killer (NK), T cells, and B cells, all of which act to control the pathogen. Monocytes release the cytokines interleukin (IL)-1β and tumor necrosis factor (TNF) and nitric oxide, in addition to chemokines that attract immature DCs. NK cells release IL-12, IL-18, and interferon gamma (IFNγ). Mature DC migrate to secondary lymphoid tissues to present *Babesia* antigens to T cells. B cells will produce antibodies against *Babesia*.

**Methods:**

In this study, we examined the transcriptional signatures of PBMCs from adult cattle (aged > 1.5 years) experimentally infected with the *B. bovis* virulent strain Vir-S74-T3Bo, during the acute phase of babesiosis, at 10 days post infection (dpi), using RNA Sequencing (RNA-Seq) technology.

**Results:**

Transcriptional signatures evident during the acute phase of babesiosis were cytokines and chemokines, such as IL-0, TNF, IL-1B, IL-18, CSF1, CXCL10 and CXCL16; pattern recognition receptors, such as CD14, TLR and NOD2; complement components, such as C1R, C2, C3aR1, CFB, CFI and CFP; cell adhesion molecules, such as ICAM1/2 and SELL; and apoptosis markers, such as CASP, BAX and BAK. We identified 1766 upregulated and 1508 downregulated genes, with fold changes ranging from two- to 429-fold. We discuss our findings in the context of immune responses to acute disease as a mechanism for adult host survival, with a focus on the molecular functions and biological processes involved in the response to *B. bovis* infection.

**Conclusions:**

In this RNA-Seq analysis, we identified genes that are up- and downregulated in response to acute *B. bovis* infection. Gene expression of IL-10, along with that of the inflammatory cytokines IL-1β, TNFα and IL-18, suggests a non-protective response to *B. bovis* at 10 dpi. These results enhance our understanding of the molecular interactions between *Babesia* and the host immune system.

**Graphical Abstract:**

**Figure:**
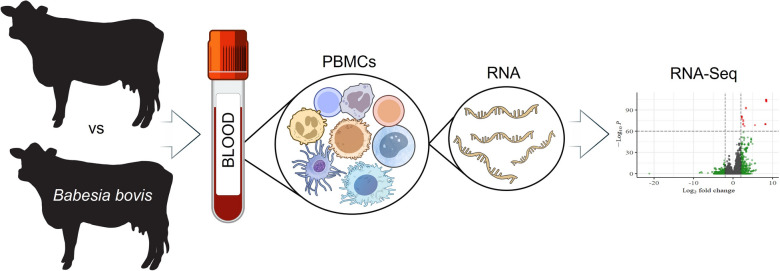

**Supplementary Information:**

The online version contains supplementary material available at 10.1186/s13071-025-07126-x.

## Background

Babesiosis is caused by tick-borne obligate intraerythrocytic pathogens of the genus *Babesia*, which belong to the phylum Apicomplexa. These parasites replicate within the red blood cells (RBCs) of a wide range of domestic and wild animals [1]. Four parasite species of the genus* Babesia*, namely* B. bovis*, *B. bigemina*, *B. divergens* and *B. major*, can infect cattle and are recognized as the causative agents of bovine babesiosis, an economically significant disease that threatens the cattle industry globally. Of these four species, *B. bovis* is considered one of the most virulent, and infection of naive cattle herds lead to high mortality [1]. The acute phase of *B. bovis*-caused bovine babesiosis typically lasts 7–14 days, and animals that survive the acute phase of the disease become persistently infected and can serve as reservoirs for *Babesia* parasite acquisition by *Rhipicephalus* spp. ticks [2].

Like other *Babesia* parasites, *B. bovis* exclusively infects the erythrocytes of its mammalian host. Upon infection, *B. bovis* triggers the activation of various host innate and adaptive immune cells, which produce molecules that may play crucial roles in protection against the invading pathogens [3]. Specifically, *B. bovis* attacks peripheral blood mononuclear cells (PBMCs), which are immune cells with a round nucleus that help protect the mammalian host against pathogens. In cattle, PBMCs include T cells (approx.  60%), B cells (approx. 25%), natural killer (NK) cells (approx.  2%), monocytes (approx. 11%), dendritic cells (DC) (approx. 1%) and innate lymphoid cells (approx. 1%) [4].

In recent years, studies focused on mammalian PBMCs have been performed to characterize protective immune responses against other apicomplexan parasites, such as *Toxoplasma gondii* [5, 6]*, Plasmodium* sp. [7, 8], *Cryptosporidium parvum* [9], *Eimeria* sp. [10] and *Theileria* sp. [11]. In addition, other studies have associated bovine PBMCs with *B*. *bovis* [12, 13] and *B. bigemina* [14, 15] infections.

The bovine innate immune response begins when free merozoites and trophozoites-infected RBCs (iRBCs) are initially recognized by monocytes, neutrophils and antigen-presenting cells (APCs). This APC recognition occurs via pathogen-associated molecular pattern (PAMP) and pattern-recognition receptor (PRR) interactions. *Babesia* PAMPs can bind to toll-like receptors (TLRs) expressed on neutrophils and monocytes [16, 17]. These phagocytic cells, in addition to NK cells, release inflammatory cytokines (interleukin [IL]-1β, IL-12, IL-18 and tumor necrosis factor alpha [TNF-α]) and nitric oxide (NO), attracting immature DCs, which in turn release IL-12 and IL-15 and phagocytose *Babesia* [18, 19]. Subsequently, mature DCs migrate to secondary lymphoid tissues (spleen and lymph nodes) and present the *Babesia* antigens to naive T cells [17]. Interferon gamma (IFNγ)-activated splenic macrophages kill the parasite by phagocytosis and by increasing the production of toxic metabolites, such as NO; they also produce IFN-α, IL-12 and IL-18 which stimulate T cells. An expansion of CD8+ T cells occurs in the spleens of infected animals [20], and in vitro assays indicate the induction and enrichment of CD4+ T cells upon contact with soluble and membrane antigens of merozoites [21]. IFN-γ produced by T cells then activate macrophages to produce more babesicidal molecules. In the chronic/persistent phase (> 2 weeks to years), B cells produce immunoglobulin (Ig)G1 and opsonizing IgG2 antibodies [17].

The resolution of acute infection in naïve animals infected with *Babesia* is thought to depend on a robust innate immune response that activates splenic macrophages via IFN-γ. This activation leads to parasite clearance through the phagocytosis of iRBCs and the production of toxic metabolites, including NO, which has been shown to possess babesiacidal activity [3, 22]. In addition to NO, specific cytokines produced by activated macrophages are important for the activation of NK and T cells, which are components of the host’s PBMC population [3]. During persistent infection, memory CD4+ T lymphocytes play a central role in the adaptative immune response [3]. Furthermore, cytokines produced by CD4 cells may be essential for immunoglobulin class switching, resulting in high-affinity and high-avidity of IgG2 [23], thereby promoting the production of specific antibodies which function as opsonins to enhance phagocytic activity [24].

Understanding the mechanisms of innate immunity during acute *B. bovis* infection, as well as understanding the adaptive immunity that control parasitemia, is critical for developing strategies to induce a protective immune response through vaccination [3]. The PBMCs together with macrophages play an important role in the innate and adaptive immune response that defend the host against *Babesia* infection and help maintain homeostasis. In this study, we evaluated the global transcriptional profile of PBMCs from adult cattle experimentally infected with a virulent strain of *B. bovis* by using high-throughput transcriptomic RNA sequencing (RNA-Seq). This technique enables bulk RNA sequencing, allowing the characterization of gene expression profiles in individual samples and the identification of molecular functions associated with differentially expressed genes in infected animals when compared to controls [25]. This, in turn, contributes to a better understanding of the role of immune or non-immune-related genes that are modulated in response to parasite infection.

## Methods

### Adult Holstein cattle and *Babesia bovis*

The animals used in this study were part of the experiments reported in Bastos et al. [26]. Three adult male Holstein cattle, aged > 1.5 years, were infected intravenously with 1 ml of a stabilate containing 10^7^ iRBCs of the virulent *B. bovis* Vir-S74-T3Bo strain, as described [26]. After infection, animals were monitored daily for clinical signs of acute bovine babesiosis, temperature and packed cell volume (PCV) until the end of the experiment, 11 days post-infection (dpi) [26].

### *Babesia bovis* parasite load in peripheral blood

Parasite load in peripheral blood was measured by quantitative PCE (qPCR), as previously described [27]. Whole blood was collected from each experimental animal before infection and at several timepoints post-infection, via jugular venipuncture into Vacutainer® tubes containing EDTA (BD Company, Franklin Lakes, NJ, USA) [26]. Genomic DNA (gDNA) was extracted from 100 µl of the whole blood samples using the QIAamp® DNA Blood Mini Kit (QIAGen, Valencia, CA, USA), following the manufacturer’s protocol. The extracted gDNA was used for qPCR to amplify the single-copy *B. bovis* msa-1 gene. For the qPCR, msa-1 primers (forward: 5′ GATGCGTTTGCACATGCTAAG 3′, reverse: 5′ CGGGTACTTCGGTGCTCTCA 3′) and probe (FAM 5′ CACGCTCAAGTAGGAAATTTTGTTAAACCTGGA 3′ TAMRA) were used. Reactions were performed under the following conditions: 95 °C for 10 min; 40 cycles of 95 °C for 30 s and 55.8 °C for 15 s; with an extension at 72 °C for 1 min [26], using a CFX96™ Real-Time PCR Detection System (Bio-Rad Laboratories, Hercules, CA, USA). The msa-1 standard curve was prepared with 10^0^ to 10^7^ copies of plasmid [27].

### Isolation of PBMCs

Peripheral blood mononuclear cells were isolated from peripheral blood using Histopaque®-1077 cell separation medium (Sigma, St. Louis, MO, USA). Briefly, the collected blood (approx. 30 ml/animal) was centrifuged at 1200 *g* for 10 min at 4 °C. After centrifugation, plasma and buffy coat were collected and layered on top of 20 ml of Histopaque®. The tube was centrifuged at 900 *g* for 30 min at 4 °C. The PBMC layer was aspirated and washed twice in 50 ml of phosphate buffered saline (PBS; without calcium or magnesium) by centrifugation at 500 *g* for 5 min. Cells were counted in a Moxi™ Z automated cell counter (ORFLO Technologies, El Cajon, CA, USA), and approximately 2 × 10^7^ cells/tube was resuspended in 300 µl of RNA later stabilization solution (Invitrogen/Thermo Fisher Scientific, Waltham, MA, USA), following which the samples were stored at − 80 °C until use.

### RNA extraction and quality control

Total RNA from PBMC samples stored in RNAlater was extracted using the miRNeasy Micro kit (Qiagen, Hilden, Germany) according to the manufacturer’s instructions. Briefly, samples were thawed on ice, the RNAlater solution was removed after centrifugation at 12,000 *g* for 5 min and RNA was extracted according to the kit protocol. RNA samples were quantified by Qubit™ 3 Fluorometer (Invitrogen/Thermo Fisher Scientific), treated with DNAse I (Invitrogen/Thermo Fisher Scientific), resuspended in 20 µl of EDTA TE (10 mM TRIS–HCl, 0.1 mM EDTA, pH 8.0; Quality Biological, Gaithersburg, MD, USA); 1 µg of the resuspended RNA per sample was submitted to the IGM Genomics Center at the University of California (San Diego, California, USA) for sequencing.

Samples from three pre-infection (0 dpi) (animal #1, animal #2, animal #3) and three post-infection (10 dpi) (animal #1, animal #2, animal #3) animals were submitted to RNA-Seq analysis. The RNA quality was assessed on a NanoDrop spectrophotometer (Thermo Fisher Scientific) at absorbances 260/280 and 260/230 nm, following the manufacturer’s recommendations. The RNA integrity number (RIN) values for pre-infection and post-infection samples were 7.0, 7.7 and 8.8 and 8.8, 8.5 and 5.9, respectively .

### RNA-Seq library preparation and sequencing

Strand-specific total RNA-Seq libraries from rRNA–depleted RNA were prepared using the TruSeq stranded total RNA library kit (Illumina, Inc., San Diego, CA, USA) and sequenced paired-end for 100 cycles each using the NovaSeq 6000 S4 PE100 high-throughput sequencing system ((Illumina, Inc.), generating an average sequencing depth of 30.5 million reads per sample.

### Identification and analysis of differentially expressed genes

Raw RNA-Seq reads were trimmed using Skewer v0.2.2 to remove adapters and low-quality bases, as previously described [28]. The trimming was performed in paired-end mode (-m mp) with a multithreaded setting (-t 32) to enhance processing speed. Trimmed reads were then aligned to the *Bos taurus* reference genome (GCF_002263795.2) using the strand-specific mapping option in the HISAT2’ alignment program (v2.2.1) [29]. The resulting SAM files were used to generate tag directories for each replicate using HOMER2’s batchMakeTagDirectory.pl script, with the -sspe and -flip arguments, as the reads were prepared with TruSeq [30]. The tag directories provided quality control metrics, such as uniquely mapped positions, total tags and fragment GC content, which were reviewed to confirm sample quality before downstream analysis.

Gene expression was quantified from the resulting tag directories using HOMER's analyzeRepeats.pl script with the *B. taurus* reference annotation (GCF_002263795.2) [31]. More specifically, raw counts (-raw) for the two treatment groups were quantified at the exon level (-count exons) using strand-specific settings (-strand +). Differential expression analysis was then conducted using the raw counts as input to DESeq2 [32]. The output included rlog-normalized counts, which were used for visualizations, and a table containing log2 fold change (LFC) and false discovery rate (FDR) values. Additional file 1: Table S1 was used to identify genes with significant changes (LFC > 1, FDR < 0.05) between 0 and 10 dpi. DESeq2 by default applies the Benjamini–Hochberg procedure for FDR adjustment.

### Gene categorization

To better understand the global transcriptomes modulated by acute *B. bovis* infection in adult cattle, we classified genes based on molecular functions, biological processes and cellular components using the Gene Ontology–AmiGO2 website (https://amigo.geneontology.org/amigo/search/) [33]. In the absence of information on genes in *B. taurus*, their ortholog in *Homo sapiens* (GCF_000001405.40) was used. The vast majority of genes analyzed by the AmiGO2 website also had evidence in the Panther (https://www.pantherdb.org/) and/or UniProtKB (https://www.uniprot.org/) websites. Genes with LFC > 1.5 were analyzed using the Metascape [34] website (http://metascape.org), based on the *H. sapiens* database, and the enriched term clusters of up- and downregulated genes were graphed.

### Validation by reverse transcription-qPCR

Five randomly chosen genes were validated by quantitative reverse transcription PCR (RT-qPCR). For this validation, total RNA (1.8 µg/reaction) extracted from each sample was reverse transcribed using a SuperScript® III kit (Invitrogen/Thermo Fisher Scientific) following the manufacturer’s recommendations. Complementary DNA (cDNA) was used as a template in the RT-qPCR analyses with the SsoFast™ EvaGreen® Supermix (Bio-Rad Laboratories), and gene expression was quantified using the CFX96™ Real-Time PCR Detection System (Bio-Rad Laboratories). The amplification reaction was performed in a final volume of 20 μl containing 1 μl of 10 μM of each primer, 7 μl of nuclease-free water, 1 μl of cDNA and 10 μl of the SsoFast™ EvaGreen® Supermix. The qPCR conditions included an initial denaturation at 95 °C for 3 min, followed by 40 cycles of denaturation at 95 °C for 30 s, annealing at 60 °C for 30 s and extension at 72 °C for 30 s. The specific primers (Additional file 2: Table S2) were designed using Primer3web software [35] and synthesized by Eurofins Genomics (Louisville, KY, USA). CFX Manager Software (Bio-Rad Laboratories) was used to obtain quantification cycle (Cq) values and to analyze expression data. The transcription level was calculated as a relative expression using the double delta Cq method [36] and is presented as the relative expression of the target gene after normalization by the reference gene glyceraldehyde-3-phosphate dehydrogenase (GAPDH). Statistical differences in gene expression levels between the *B. bovis*-infected and control groups were analyzed using a paired t test in Prism software. A *P*-value < 0.05 was considered to be statistically significant.

## Results

**Fig. 1:**
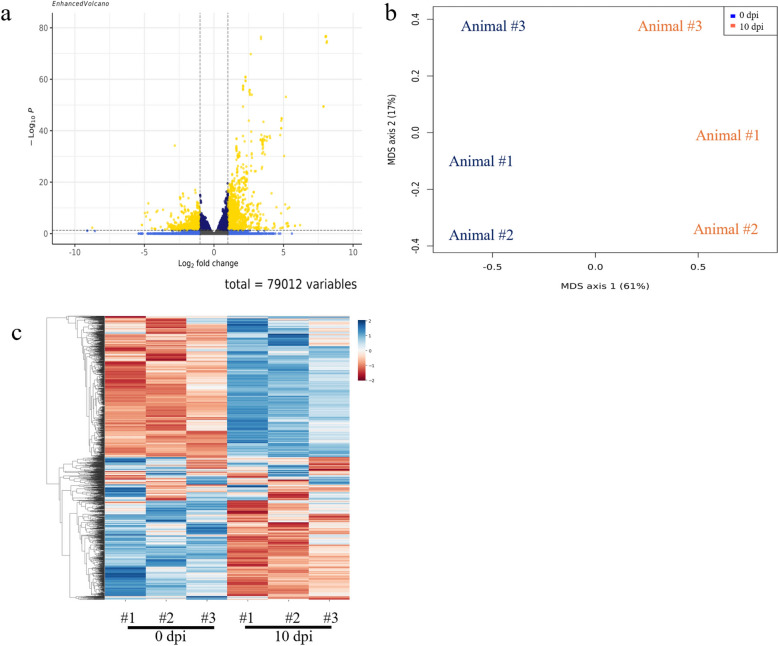
**a** Volcano plot of all genes with cutoffs log2 fold change (LFC) > 1 and false discovery rate (FDR) (adjusted *P*-value) < 0.05. Non-significant genes are represented in gray, genes significant only for LFC are given in light blue, genes significant only for FDR are represented in royal blue and genes significant for both LFC and FDR are represented in yellow. Left side shows genes downregulated in 10 dpi and right side shows genes is upregulated in 10 dpi. **b** MDS plot of the top 10,000 genes. **c **Heatmap of a subset of rlog normalized counts for each replicate (animal #1, animal #2 and animal #3 for 0 and 10 dpi) was selected based of the top 10,000 variable transcripts and plotted into a cluster map with rows z-score scaled. dpi, Days post-infection; MDS, multidimensional scaling

**Fig. 2:**
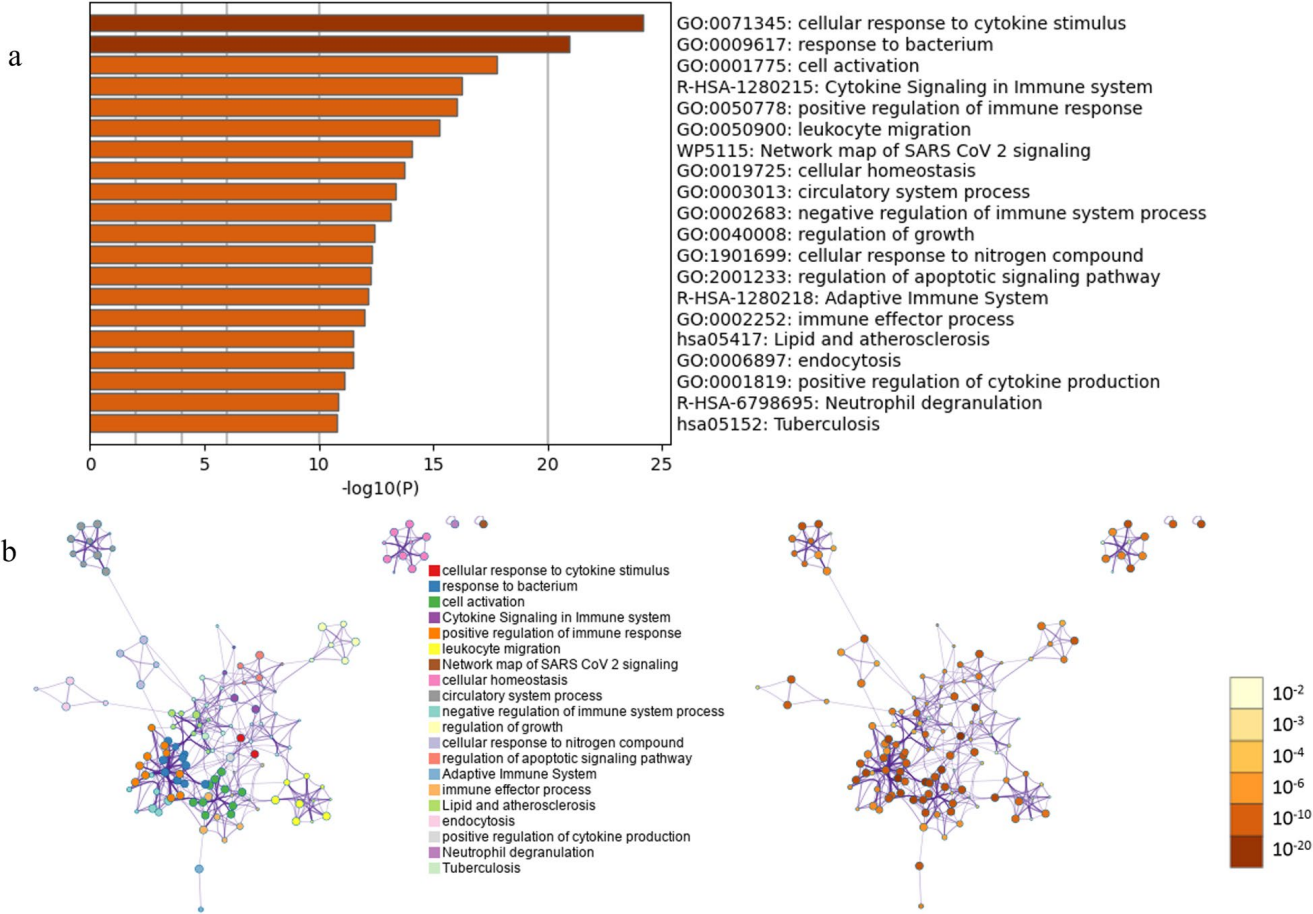
**a** Top 20 enriched terms clusters in upregulated genes based on the *Homo sapiens* database by Metascape analyses. **b** Network of enriched terms by cluster ID and a significant *P*-value. Only genes with log2 fold change > 1.5 and false discovery rate < 0.05 were included in the analysis. GO, Gene Ontology. All figures were created by http://metascape.org

**Fig. 3:**
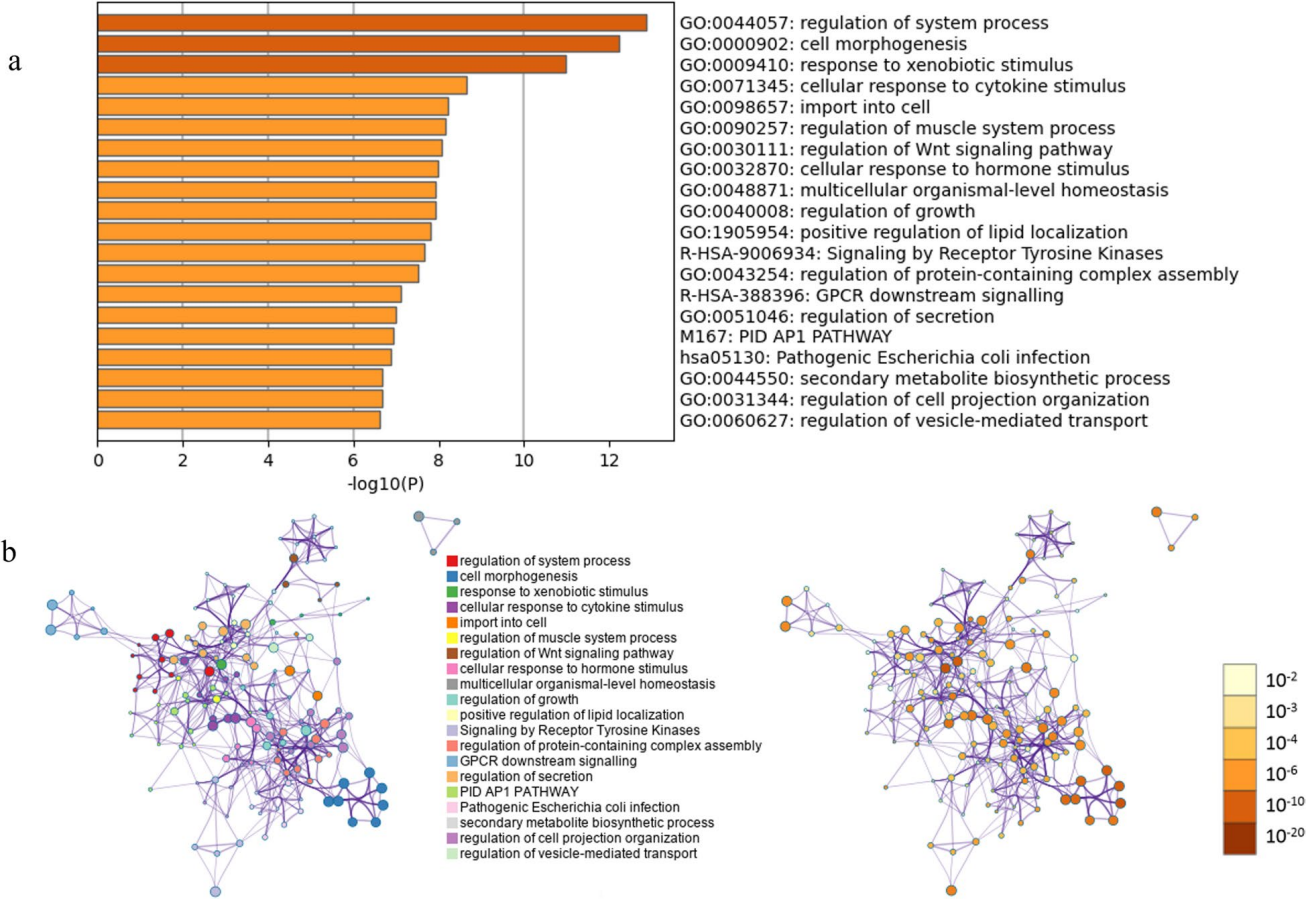
**a** Top 20 enriched terms clusters in downregulated genes based on the *Homo sapiens* database by Metascape analyses. **b** Network of enriched terms by cluster ID and a significant *P*-value. Only genes with log2 fold change > − 1.5 and false discovery rate < 0.05 were included in the analysis. GO, Gene Ontology. All figures were created by http://metascape.org

A total of 79,012 transcripts were found in this study, including all annotated transcript types present in the *B. taurus* reference annotation (GCF_002263795.2), of which about 85% were messenger RNA (mRNA) related to and 15% associated with non-coding RNAs such as lncRNAs, pseudogenes, snRNAs and other annotated biotypes (Additional file 3: Table S3). Of these 79,012 transcripts, 1766 were upregulated (LFC > 1, FDR < 0.05), with the changes ranging from two- to 278.8-fold, and 1508 were downregulated (LFC < −1, FDR < 0.05), with changes ranging from two- to 429.6-fold, in *B. bovis*-infected animals after 10 dpi (Additional file 1: Table S1). The numbers of raw reads, trimmed reads and alignment rate of each sample analyzed in this RNA-Seq are described in Additional file 4: Table S4. The total number of differentially expressed transcripts in response to *B. bovis* infection is shown in the volcano plot in Fig. 1a. Multidimensional scaling (MDS) analysis separated the biological replicates into two distinct groups corresponding to 0 and 10 dpi, respectively (Fig. 1b), and a heatmap shows the overall expression of transcripts for all six replicates (Fig. 1c). The most abundant Gene Ontology (GO) biological processes terms for the up- and downregulated genes, along with the network of enriched terms by cluster ID and their* P*-values were generated and are presented in Fig. 2a, b and 3a, b.

**Fig. 4:**
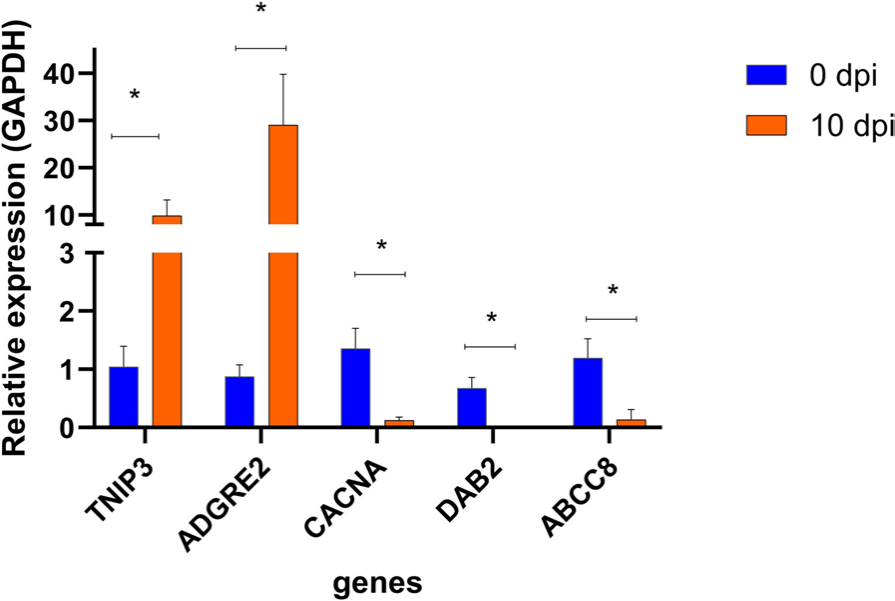
Relative expression of non-infected (0 dpi) and *Babesia bovis*-infected peripheral blood mononuclear cells (10 dpi) complementary DNA (cDNA) were used as a template in the quantitative reverse transcription PCR (RT-qPCR) assay. cDNA from animal #1, animal #2 and animal #3 on 0 dpi constituted the control samples (in blue), and DNA from animal #1, animal #2 and animal #3 on 10 dpi constituted the *B. bovis*-infected (in orange) samples. Genes: *TNIP3*, TNFAIP3 interacting protein 3; *ADGRE2*, adhesion G protein-coupled receptor E2; *CACNA*, calcium channel, voltage-dependent, alpha 2/delta subunit 1; *DAB2* clathrin adaptor protein; *ABCC8* ATP binding cassette subfamily C member 8; glyceraldehyde-3-phosphate dehydrogenase (*GAPDH*) was used for RT-qPCR normalization. The paired t-test was performed in Prism software. Asterisk indicates significance at *P < 0.05. dpi, Days post-infection

To validate the RNA-Seq, RT-qPCR was performed to determine expression levels of five genes identified from PBMCs responding to *B. bovis* infection. The RT-qPCR data corroborated the high-throughput RNA-Seq results. Relative gene expressions of TNIP3 and ADGRE2 were significantly higher in the infected group compared to the control group (Fig. 4). Relative gene expressions of the CACNA, DAB2 and ABCC8 genes were significantly lower in *B. bovis*-infected animals compared to the uninfected control group (Fig. 4).

**Fig. 5:**
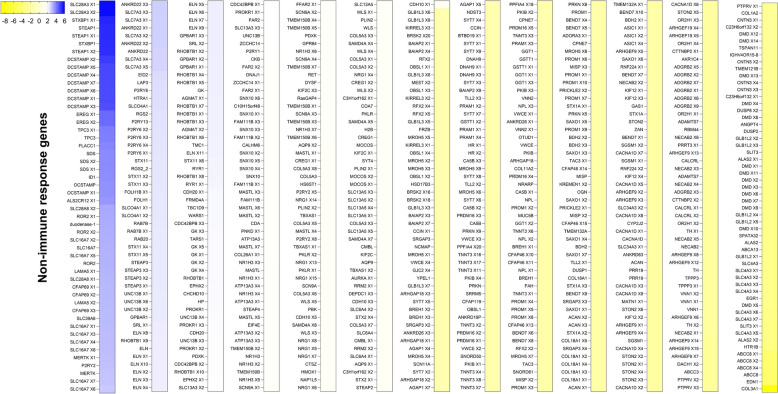
Bovine non-immune response genes associated with *Babesia bovis* infection at 10 days post-infection, compared with uninfected animals. The intensity of the color from yellow to blue indicates the magnitude of the log2 fold change (LFC), based on the color scale at the top of the figure and plotted into a cluster map with rows z-score scaled. Only genes with LFC > 1.5 and false discovery rate < 0.05 were included in the analysis. The full names of the abbreviated genes are given in Additional file 1: Table S1

**Fig. 6:**
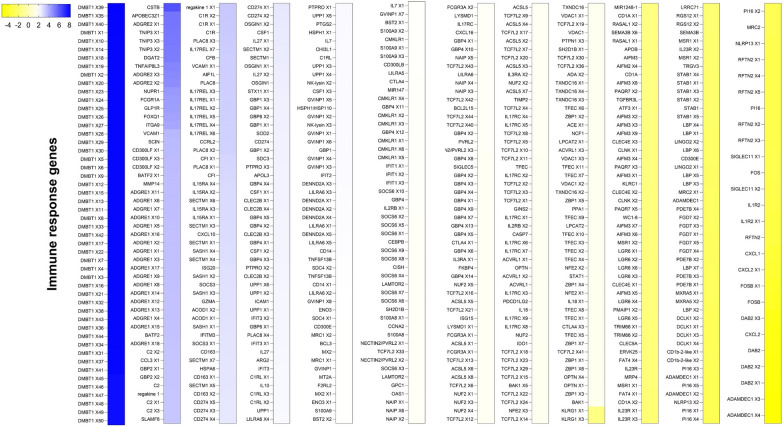
Bovine immune genes associated with *Babesia bovis* infection at 10 dpi, compared with uninfected animals. The intensity of the color from yellow to blue indicates the magnitude of the log2 fold change (LFC), based on the color scale at the top of the figure and plotted into a cluster map with rows z-score scaled. Only genes with LFC > 1.5 and false discovery rate < 0.05 were included in the analysis. The full names of the abbreviated genes are in Additional file 1: Table S1. dpi, Days post-infection; PBMCs, peripheral blood mononuclear cells; RBCs, red blood cells

In this study, genes modulated in response to *Babesia* infection were classified as non-immune genes (Fig. 5; Additional file 5: Figure S1) or immune genes (Fig. 6; Additional file 6: Figure S2). Of the genes classified as non-immune, a large group were involved in molecular transport, metabolism, calcium channel activity, cellular components, cell surface molecules (such as ABCs transporters), metalloreductases (such as STEAPs) and cell signaling (such as Wnt pathway), and others were modulated by *B. bovis* infection (Fig. 5; Additional file 5: Figure S1).

**Fig. 7:**
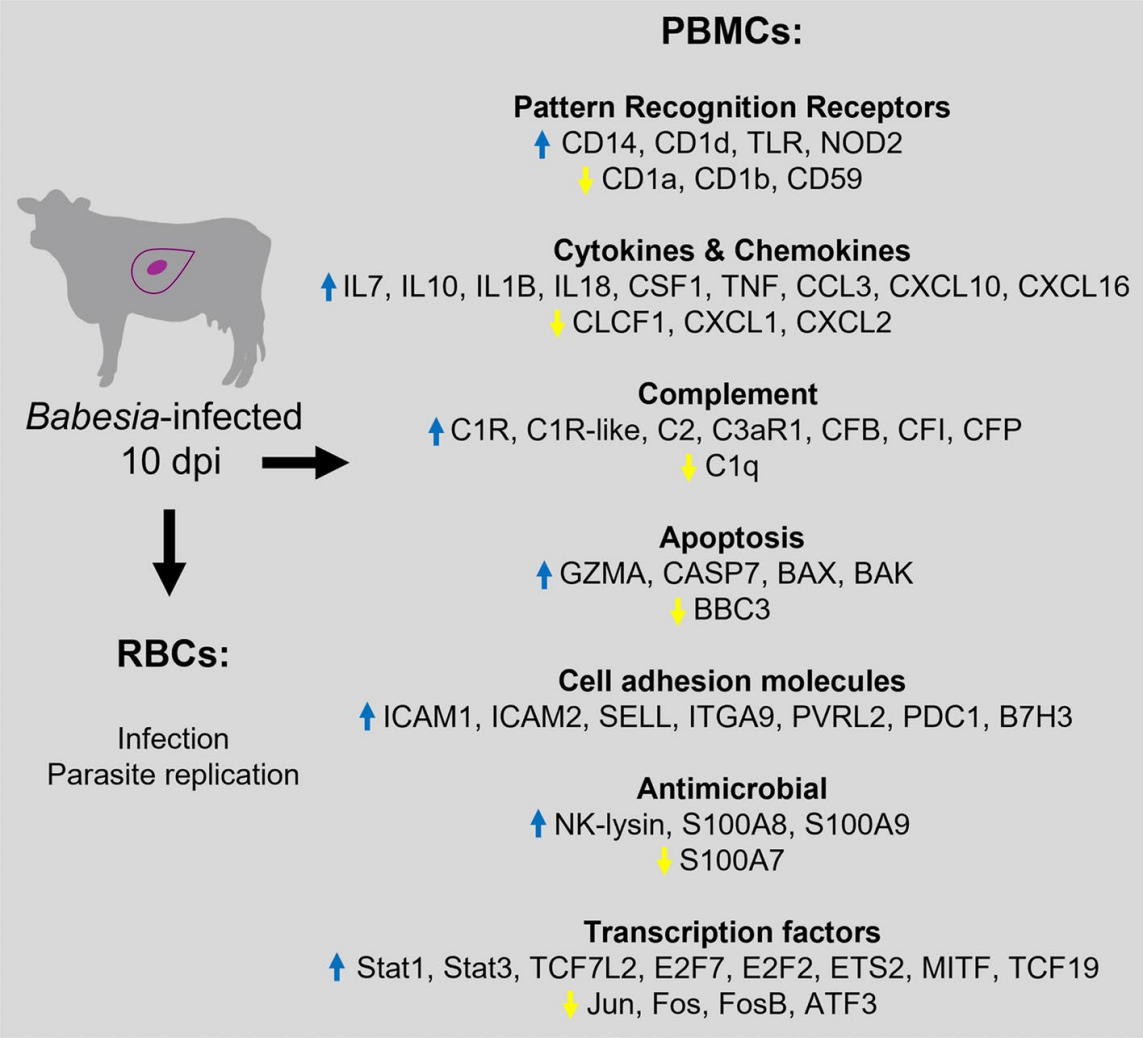
Schematic summary of bovine gene modulation in response to *Babesia bovis* infection or in response to the acute phase of babesiosis. The blue arrow indicates that genes were upregulated, and the yellow arrow indicates that genes were downregulated

Immune genes were also modulated in response to *B. bovis* infection, including those for cell surface molecules, inflammatory response, cellular components, complement, cytokines and chemokine production, cell signaling pathways and apoptosis (Fig. 6; Additional file 6: Figure S2). Figure 7 provides a summary that identifies potentially important bovine genes in response to *B. bovis* infection.

## Discussion

### Immune cell surface molecules

Among the immune genes assessed, several surface molecules, such as CD163, CD274, CD300LB, CD1d and CD14, were upregulated in the infected animals, with fold changes ranging from 2.2 to 6.2. In addition, CD1a, CD1b-2like, CD276, CD59 and CD300E transcripts were downregulated by 2.1- to 6.3-fold in infected animals. CD163, also known as hemoglobin scavenger receptor, is a monocyte/macrophage-specific receptor whose expression can be upregulated by glucocorticoid, IL-10, IL-6 and heme/hemoglobin [37]. The expression of this gene has also been found to be upregulated in adult cattle infected with a virulent strain of *B. bigemina* [15] and in humans with malaria [38]. CD274, also known as programmed death-ligand 1 (PD-L1), an immune molecule widely expressed in mononuclear phagocytes, regulates T cell proliferation, IL-10 production and inflammatory responses [39–41]. The CD300 family of molecules modulates a wide range of immune cell processes and is also involved in efferocytosis [42]. Mouse CD300LB, like CD14, appears to be implicated in TLR4 activation on immune cells following lipopolysaccharide (LPS) binding [43]. In addition, CD300LB is expressed in myeloid cells and recognizes phosphatidylserine on apoptotic cells. In conjunction with F-actin, CD300LB appears to facilitate the engulfment of apoptotic cells by activating the PI3K-Akt kinase pathway in macrophages [44]. In humans, CD14 on monocytes is a PRR that binds directly to LPS and contributes to the activation of innate immune responses, particularly through TRL4 signaling [45]. This molecule plays a role in inflammation and metabolic diseases [45]. In *B. taurus*, CD14 seems to have a role in inflammatory response with positive regulation of type I and II IFN, IL-8 and TNF production. Interestingly, seven lipopolysaccharide-binding protein (LBP) variants were downregulated 4.8- to sixfold in *B. bovis*-infected animals. The role of LBP is to bind to bacterial LPS to present it to CD14 and TLR4 [46]. Another molecule downregulated by infection was CD1-b, which recognizes various lipids—including microbial lipid antigens—and is present on T and NK cells [47]. CD59 can regulate the formation of C8α and C9 binding during the assembly of the complement membrane attack complex (MAC) [48]. MAC is a cytolytic effector of the immune system that forms pores into the lipid bilayer of the plasma membrane of pathogens or target cells, generated through sequential assembly from the soluble complement proteins, leading to osmolysis [49].

The chemerin chemokine-like receptor 1 (CMLKR1) transcripts were upregulated 3.6-fold in *B. bovis*-infected animals. In humans, the expression of CMKLR1 has been detected in macrophages and neutrophils [50], and its upregulation was identified in response to a high concentration of LPS [51]. The activation of CMLKR1 facilitates both pro-inflammatory and anti-inflammatory effects, depending on the physiological contexts [50]. Zhang and collaborators reported that CMLKR1 activation during inflammation processes is subject to spatiotemporal regulation. During the early stage of inflammation, CMKLR1 signaling is primarily pro-inflammatory, promoting chemotaxis and the activation of DCs and macrophages. In contrast, during the later stage of inflammation, CMKLR1 activation can initiate pro-resolution pathways that help reduce inflammation [50]. Sialic acid-binding immunoglobulin-like lectin (SIGLEC) 5, a type 1 membrane protein containing an amino-terminal V-set immunoglobulin domain that mediates sialic acid recognition [52], was upregulated 3.2-fold in *B. bovis*-infected animals. SIGLEC5 is expressed in neutrophils, monocytes and B cells and contains tyrosine-based signaling motifs—particularly immunoreceptor tyrosine-based inhibitory motifs (ITIMs), which are involved in cell signaling and endocytosis [52].

The mannose receptor C (MRC) 1 variants were upregulated fourfold, while the MCR2 genes were downregulated 5.2- and 7.3-fold in the *B. bovis*-infected animals. MRC can bind to and internalize a variety of endogenous and pathogen-associated ligands, including microbial surface glycan structures [53]. Once endocytosed, antigens are directed toward cross-presentation on major histocompatibility complex (MHC) molecules, leading to the activation of CD8+ T cells. Additionally, MRC can interact with the TLR2 signaling pathway [53]. The LRRC71 gene was downregulated 3.8-fold in *B. bovis*-infected animals. This gene encodes a leucine rich repeat (LRR) protein and has been shown to be involved in mammalian host defense (TLR pathway), where it detects PAMPs and activates the immune system [54].

Three adhesion G protein-coupled receptor E2 (ADGRE2) variants were upregulated by 27-, 30- and 40-fold in the three infected animals. ADGRs are considered to be massive signaling platforms that are crucial for the integration of adhesive, mechanosensory and chemical stimuli [55]. ADGRE2 encodes a member of the epidermal growth factor–seven transmembrane subclass of adhesion G-protein coupled receptors. It is expressed predominantly in myeloid leukocytes, with the highest expression in neutrophils and macrophages [56].

The Fc fragment of the IgG receptor Ia (FCGR1A) and the low affinity Fc fragment of IgG IIIa receptor (FCGR3A) were upregulated three- to 20-fold during acute *B. bovis* infection. Fc receptors play an important role in immune response, as they mediate the specific recognition of various types of antigens by leucocytes [57]. FCGR1A is a high-affinity receptor for monomeric IgG, whereas FCGR3A exhibits low affinity for monomeric IgG. However, these molecules can bind aggregated IgG through low-affinity, high-avidity multimeric interactions. This feature is important in the recognition and binding of antibody–antigen complexes and can trigger several immunoregulatory functions, including degranulation, phagocytosis and regulation of antibody production [57].

Genes encoding cell adhesion molecules (CAM) from different categories were modulated in response to *B. bovis*. Among these, intercellular adhesion molecule (ICAM) 1 and 2, vascular cell adhesion molecule 1 (VCAM1), selectin L (SELL), integrin alpha 9 (ITGA9), cadherins (CDH10 and CDH20), immunoglobulin superfamily (IGSF6) and nectin cell adhesion molecule 2 (NECTIN2 or PVRL2) genes were upregulated two- to 17.6-fold and CD276 (or B7/H3) molecule and cadherin (CDH24 and CDH 3) transcripts were downregulated 2.1- to 2.6-fold in *B. bovis*-infected animals. CAMs are expressed on the cell surface and play a role in immune response and inflammation, along with a wide range of other biological processes.

Overall, we identified host receptors that appear to be important for the recognition of PAMPs. Many of these are described in the literature as being responsive to LPS, which is a constituent of the bacterial wall. There are no reports on why *Babesia* causes this modulation of LPS-related genes, and *Babesia* does not contain LPS. Many of the genes modulated by LPS exposure in bovine PBMCs [4] were also modulated by *B. bovis*, such as CD14, CXCL2, CXCL10, MMP14, ISG15, ISG20, S100A8, CD274, PLAC8, GBP5, GBP4, IFITM3, SOCS3, FCGR1A, CFB, CSF1 and others [4]. Interestingly, it has been reported that peritoneal macrophages from *Babesia microti*-infected mice exhibited hyperreactivity and released cytokines in response to LPS [58]. The actual *Babesia* spp. PAMPs recognized by the host immune system remain unknown, and it appears that the glycosylphosphatidylinositol anchor (GPI) in the parasite cell membrane may be a candidate, as described for *Plasmodium falciparum* and *Trypanosoma cruzi* [59]. In addition, *Babesia* spp. carry CpG motifs in their DNA [60], which may also act as PAMPs that bind to receptors to activate innate immune responses.

### Complement

 Sixteen cattle complement genes, including complement C2, complement factor B (CFB), complement C1r (C1R), C1R-like, C3aR1, complement factor properdin (CFP) and complement factor I (CFI) were upregulated two- to 10.6-fold in response to the infection. Two C1qA chain transcripts showed a twofold downregulation by the infection. The complement system consists of several plasma proteins that work together to opsonize microorganisms and initiate a series of inflammatory responses that aid in fighting infection [61]. A study demonstrated the depletion of complement proteins C2, C3, C4 and C5 during *Babesia rodhaini* infection in a rat model, suggesting that the complement pathways are activated during the protozoan infection [62]. However, the hypocomplementemia may be related to the anemia and thrombocytopenia observed in this disease, as well as to the formation of antigen–antibody complexes associated with C3, which have been detected in the renal glomeruli of infected rats [62]. Additional studies involving serum protein quantification of complement components should be conducted in *B. bovis*-infected cattle to better determine whether the complement pathways are activated during infection.

### Cytokines and chemokines

**Fig. 8:**
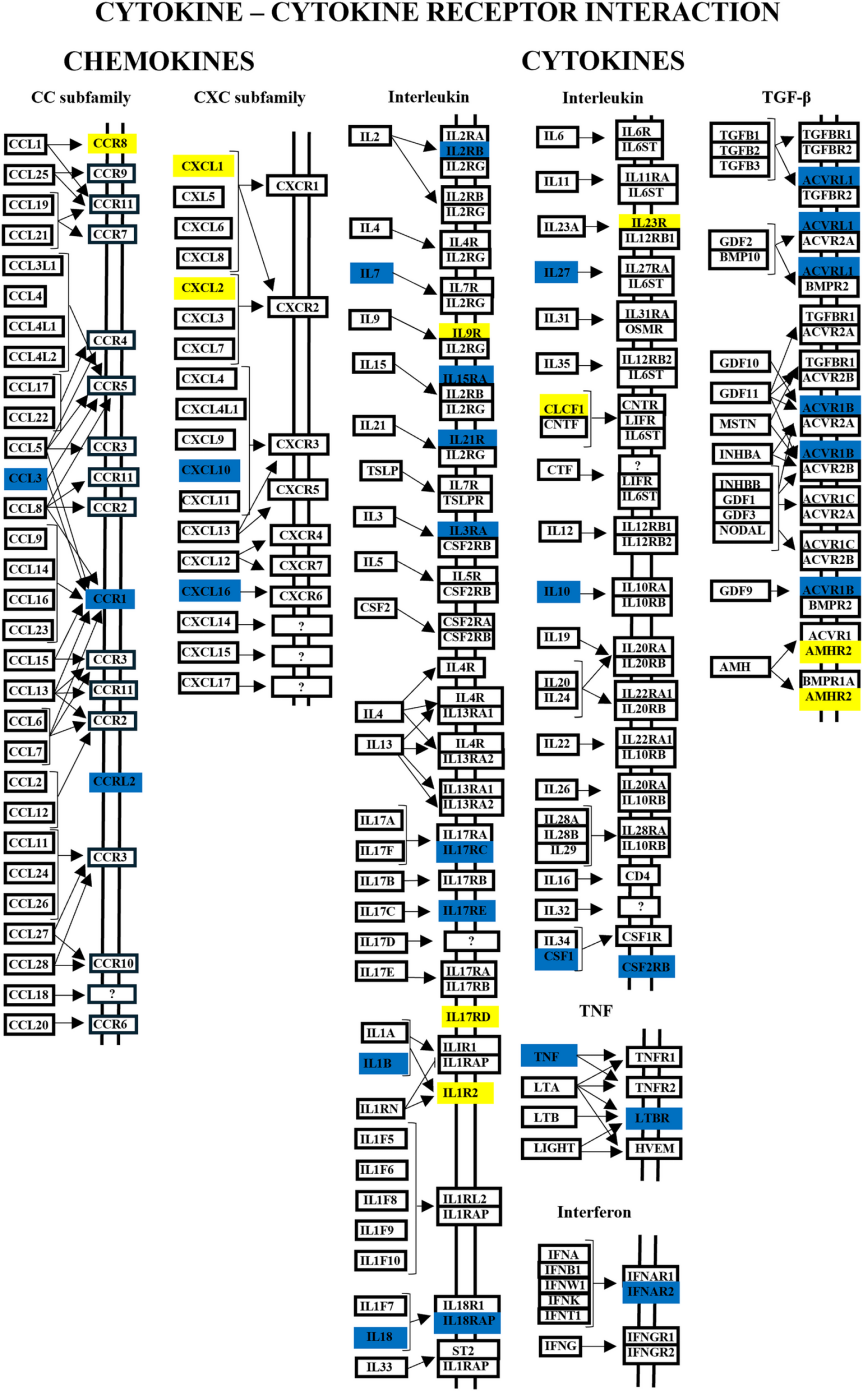
Bovine cytokine and chemokine genes and their interactions with receptors, modulated in response to *Babesia bovis* infection or as a result of the acute phase of the disease at 10 days post-infection. The scheme was used from KEGG PATHWAY Database and modified. Upregulated genes are highlighted in blue, downregulated genes are highlighted in yellow and non-modulated genes are in black font. IL, Interleukin; TGFβ, transforming growth factor beta; TNF, tumor necrosis factor

 Interleukin-10, an anti-inflammatory mediator that helps protect the host from exacerbated responses to pathogens [63], was upregulated 4.5-fold in the *B. bovis*-infected animals. IL-10 is a secreted cytokine produced by cells of the innate immune system, including DCs, NK cells, eosinophils, neutrophils, macrophages and mast cells. It is also expressed by various cells of the adaptive immune system, including T cells (TH1, TH2 and TH17 cell subsets, CD8+ T cells) and B cells (review by [64]). Studies on *Leishmania* have reported that IL-10 inhibits the killing of intracellular pathogens by reducing the production of NO metabolites via IFN-γ-activated macrophages [65, 66]. In humans, IL-10 also inhibits the antigen-presenting capacity of monocytes through by downregulating class II MHC expression, thereby preventing the antigen-specific T cell proliferation [67]. Additionally, receptors for the anti-inflammatory transforming growth factor (TGF) family ACVRL1, ACVR1B and LTBR were upregulated 2.1- to threefold in infected animals. In contrast, the pro-inflammatory cytokines IL-1B, IL-18, IL-27 and TNF were upregulated by 2.1- to 5.5-fold in infected animals. Unlike young animals, which typically recover from *B. bovis* infection through IL-12-mediated inflammatory response [68], in the present study we found no evidence of IL-12 modulation in adult animals. Moreover, nine transcript-associated receptors for IL-1, IL-9, IL-17 and IL-23 (IL1R2, IL9R, IL17RD and IL23R) were downregulated by 2.1- to 10.1-fold in *B. bovis*-infected animals. Figure 8 presents a schematic of cytokines and chemokines and their receptor interactions.

Four colony-stimulating factor 1 (CSF1) variants were upregulated by 4.2- to 5.6-fold and two regakine 1 genes were upregulated 9.8- and 10.2-fold, in *B. bovis*-infected cattle. CSF-1 is a glycoprotein also known as macrophage-CSF (M-CSF). CSF-1 and its receptor control the survival, proliferation and differentiation of mononuclear phagocytes to fully differentiated nondividing macrophages [69]. Regakine is a plasma chemoattractant chemokine that is constitutively present in bovine serum and works synergistically with IL-8 to attract neutrophils, thereby amplifying the inflammatory response [70, 71]. Chemokine ligand 1 (CXCL1) and 2 (CXCL2) were downregulated by 12.6- and 26.3-fold in the *B. bovis* infected cattle. The CXCL10 transcript was upregulated 7.1-fold in the infected animals, reinforcing previous observations [26], and CXCL16 was also upregulated 3.3-fold in infected animals. CXCL1 is expressed on the surface of neutrophils, NK cells and macrophages and has neutrophil chemoattractant activity [72]. CXCL2 is capable of recruiting monocytes, memory T cells and DCs [73]. CXCL10 is an IFN-γ-induced protein and is produced by macrophages, T cells and other cell types [74]. The chemokine CCL3 and its receptor CCR1 were upregulated 10.5- and two-fold, respectively, by the infection (Fig. 8).

IFN-γ, the cytokine reported in the literature as important for the initial control of *B. bovis* by the host, was not detected in this transcriptome at 10 dpi. However, IFN-γ was detected by an enzyme-linked immunosorbent (ELISA) analysis in the sera of animals infected with *B. bovis* at different times in a previous study [26]. These discrepant results may suggest that IFN-γ is produced by splenic cells but not by PBMCs, or that IFN-γ production in PBMCs occurs at different timepoints that were not analyzed in this transcriptomic analysis. Similar to the severe manifestations of malaria (*Plasmodium* spp.) in susceptible mice, the lethality of bovine babesiosis in adult animals appears to be associated with the nature and regulation of cytokine responses. The inadequate development of inflammatory responses also seems to contribute to severe infections and mortality among *B. bovis*-infected hosts [75].

### Signaling pathways

Some genes identified in the present RNA-Seq analysis are involved in cell signaling pathways—for example, TLR8 and toll-like receptor adaptor molecule 2 (TICAM2) were upregulated 2.1- to 2.3-fold in infected animals. DENN domain-containing 2A (DENND2A) transcript was upregulated fourfold during *B. bovis* infection. DENND2A interacts with SAM and SH3 domain-containing protein 1 (SASH1), a scaffold protein involved in TRL signaling, and inhibits the NF-κB activation through this pathway, thereby suppressing the production of proinflammatory cytokines and interferons [76]. In addition, in this study, SASH1 genes were upregulated by 6.6-fold in *B. bovis*-infected animals. The basic leucine zipper transcription factor 2, ATF-like (BATF-2) gene was upregulated by 11-fold in *B. bovis*-infected animals. BATF, a member of the AP-1 family, forms heterodimers with Jun subunits and inhibits AP-1-mediated transactivation. BATF expression is largely restricted to lymphoid organs and is upregulated in T and NK cells upon stimulation [77]. A previous study linked BATF-2 to the control of *T. cruzi* infection in mice and demonstrated a novel function of BATF-2 as a negative regulator of IL-23 in innate immune cells [78]. IL-23 contributes to *T. cruzi*-specific IL-17 production, and its exacerbated levels have been implicated in chronic tissue inflammation [78]. The disabled-2 (DAB2) gene was downregulated by 24- to 31.4-fold in infected animals. DAB2 plays a role in signaling pathways involved in cell differentiation, proliferation, migration, tumor suppression and cellular homeostatic mechanisms [79]. It can suppress inflammatory responses, mainly by suppressing TLR signaling in APCs [79]. Three other AP-1 transcription factor subunits—JUN, FOS and FOSB—were downregulated by 2.5- to 17.6-fold in *B. bovis*-infected animals.

Several components of the Jak/Stat pathway, including the inhibitors SOCS and the transcription factor STAT, were also modulated by the infection. Suppressors of cytokine signaling (SOCS) 1, 3 and 6 genes were upregulated 2.5- to 6.4-fold in infected animals. The STAT1 and -3 transcription factor genes were upregulated two- to 2.8-fold in infected animals. STAT1 expression has also been found to be upregulated in animals infected with a virulent strain of *B. bigemina* [15]. The upregulation of expression of the inhibitors SOCS1 and SOCS3 of the Jak/Stat pathway and downregulation of the transcription factors JUN and FOS of the Jnk pathway, may suggest that these pathways are depressed in response to *B. bovis*.

An antimicrobial peptide (AMP) NK-lysin transcript was upregulated by 4.2-fold in infected animals. This AMP is classified as cationic and amphipathic α-helical [80]. NK-lysin produced by T and NK cells has been shown to be effective against various microbes, including multidrug-resistant *Salmonella* isolates [80]. S100 calcium-binding protein A8/9/12 transcripts were upregulated 2.5- to 3.8-fold in infected animals. These proteins chelate metallic nutrients, directly inhibiting the growth of pathogens in the host [81].

In malaria blood-stage infection, parasite DNA, RNA and GPI anchors interact with TLR9, TLR7 and TLR2, respectively. Additionally, microparticles released from iRBCs and heme produced during infection can activate TLR4, triggering the MAPK and NF-κB signaling pathways and inducing the production of cytokine and chemokine [82]. *Plasmodium* RNA can activate the MAVS-TBK1-IRF3/IRF7 signaling pathway; however, this activation appears to induce the expression of SOCS1, which negatively regulates TLR7-mediated RNA-induced type I IFN production [83]. In addition, *Plasmodium* hemozoin and uric acid can activate the NLRP3 and NLRP12 inflammasomes, leading to the cleavage of pro-caspase 1 and activation of caspase 1 [84]. In the present study, we identified a modulation of molecules involved in the NF-κB signaling, suggesting that *B. bovis* PAMPs may be recognized by this host pathway, similar to what occurs in malaria. However, based solely on the transcriptional analysis of signaling pathway components, we were unable to confirm or refute the hypothesis that the NF-κB, Jnk, and Jak/Stat pathways are activated during *B. bovis* infection. A more comprehensive understanding of the pathways regulating the protozoan parasite's response is required.

### Immunometabolism

In *B. bovis*-infected animals, transcripts of aconitate decarboxylase 1 (ACOD1), a regulator of immunometabolism in inflammation and infection, were upregulated by 6.4-fold. The upregulation of ACOD1 occurs in activated immune cells in response to pathogen infection, PAMPs, inflammatory cytokines, hormones and damage-associated molecular patterns [85]. The activation of the ACOD1 pathway may limit pathogen infection, but abnormal ACOD1 expression can lead to tumor progression, neurodegenerative disease and immune paralysis [85]. Syndecan (SDC) 3 and SDC4 genes were upregulated by 4.9- and fourfold in infected animals. SDCs are proteoglycans that are part of the glycocalyx on the surface of various cell types [86]. SDC is involved in the adhesion, migration, proliferation and cellular differentiation in cell signaling pathways and plays a major role in inflammation by regulating leukocyte extravasation and cytokine function [87, 88]. Eight variants of secreted and transmembrane 1-like (SECTM1) transcript were upregulated by 5.5- to 8.2-fold in *B. bovis*-infected animals. In mice, SECTM1 functions as a ligand for T cells [89], and both soluble and cell surface-anchored SECTM1 have been shown to activate CD4+ and CD8+ T cells [89]. SECTM1 can also act as a T cell costimulator, enhancing IL-2 production and proliferation [89]. Uridine phosphorylase 1 (UPP-1) genes were upregulated 4.5-fold during *B. bovis* infection. UPP is an enzyme in the pyrimidine pathway, catalyzing the reversible phosphorolysis of uridine to uracil and ribose-1-phosphate (or deoxyribose‐1‐phosphate) [90]. Additionally, the protein tyrosine phosphatase receptor type O (PTPRO) genes were upregulated 4.6-fold in infected animals. PTPRO genes are expressed by hematopoietic cells and may play important roles in many cellular processes such as growth, differentiation, activation and immune responses [91].

An allograft inflammatory factor 1 (AIF1) and allograft inflammatory factor 1-like (AIF1L) transcripts were upregulated 2.4- to 9-fold in infected animals. AIF1 is a calcium-binding protein produced by lymphocytes, macrophages and monocytes [92], and it also binds to actin filaments. In humans, AIF-1L is involved in inflammation responses triggered by pathogenic infection or tissue injury, and increased AIF1 expression has been linked to several diseases, including endometriosis, breast cancer, atherosclerosis, rheumatoid arthritis and fibrosis (review by [92]).

### Apoptosis

**Fig. 9:**
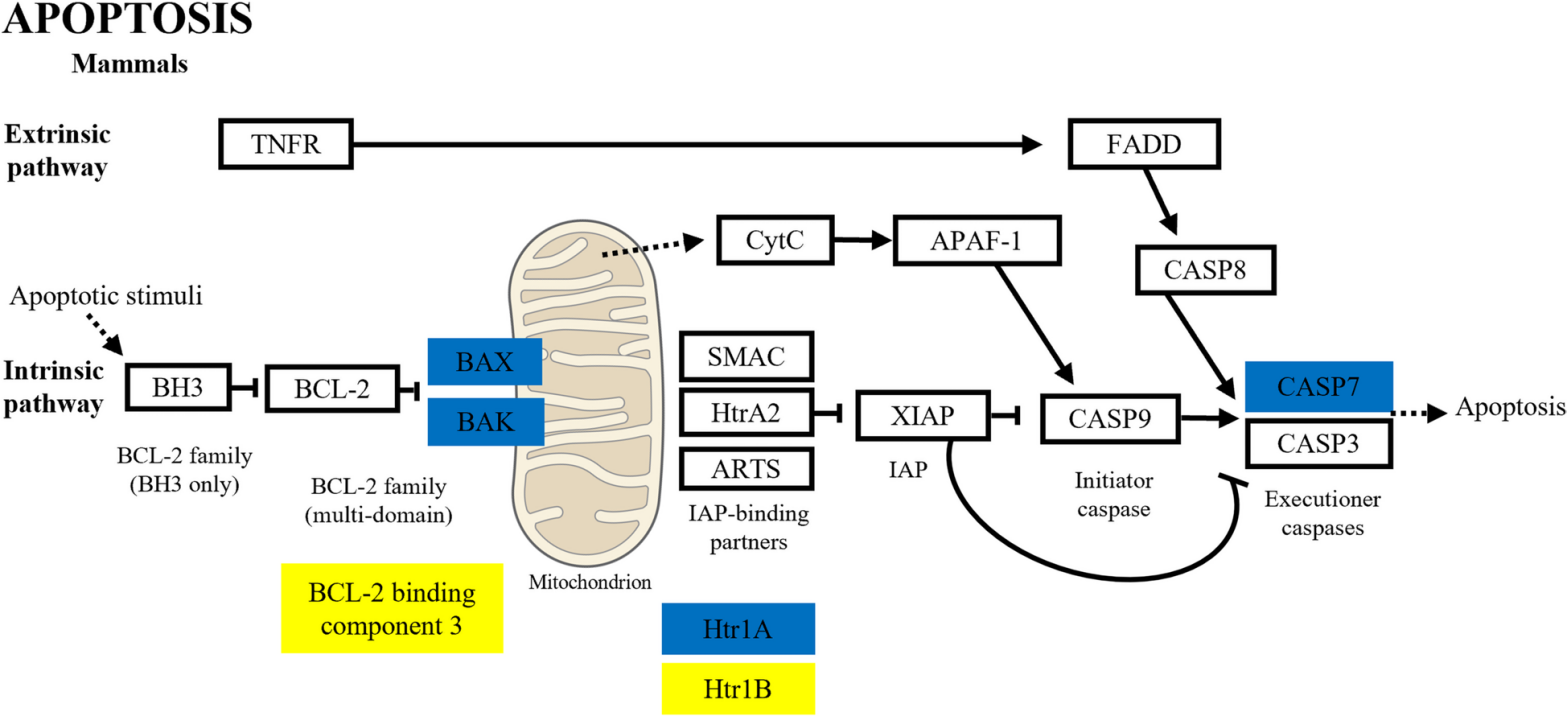
Apoptosis pathway genes, modulated in response to *Babesia bovis* infection or as a result of the acute phase of the disease at 10 days post-infection. The scheme was used from KEGG PATHWAY Database and modified. Upregulated genes are highlighted in blue, downregulated genes are highlighted in yellow and non-modulated genes in black font

 In this study, we observed a decrease in white blood cells, lymphocytes and neutrophils in the peripheral blood of animals acutely infected with *B. bovis* [26]. In the transcriptome, some molecules associated with apoptosis were identified. Granzyme (GZM) A genes were upregulated 6.4-fold in *B. bovis*-infected animals. GZMA activates the mitochondrial pathway of programmed cell death, leading to the generation of reactive oxygen species (ROS), which in turn initiates DNA damage in the nucleus [93]. During the attack on target cells, GZMA are released into the target cytosol by cytotoxic T cells and NK, inducing a rapid increase in intracellular ROS. This elevated ROS level is sustained for at least 2 h, ultimately triggering cell death [94]. However, the authors of a previous study reported that there is an increase in the number of parasites during the acute phase of bovine babesiosis in adult animals [26], suggesting that *B. bovis* evades the host’s immune system. Furthermore, we observed that NO expression was not modulated in bovine PBMCs after 10 dpi. Additionally, there are reports in the literature that cytokine IL-12 can induce the expression of GZMA, which, in turn, can induce the production of other pro-inflammatory cytokines such as TNF. GZMA can also increase the conversion of pro-IL1β into active mature IL1β, functioning as a regulator of pro-inflammatory responses [95], corroborating the results seen in this study. In addition, the expression of caspase 7 (Casp7) was upregulated threefold in infected animals. In the intrinsic pathway of apoptosis, BAX and BAK genes were upregulated two- to 2.8-fold, and one BCL-2-binding component 3 was downregulated 3.5-fold in infected animals. These results suggest that the apoptosis pathway (Fig. 9) is possibly active and is responsible for the decrease in cell numbers that Bastos and collaborators observed in these animals [26]. However, further studies are needed to determine whether the upregulation of GZMA, Casp7, BAX and BAK can be involved in apoptosis of white blood cells in *B. bovis*-infected cattle.

### Study limitations

 Although this study generated robust data that enhances our understanding of the acute phase of infection in adult animals, it has some limitations. Notably, the RNA-Seq technique used analyzes global transcriptomic expression from a mixed cell population, which makes it difficult to attribute the identified gene expression patterns to specific cell types. To address this, we plan to use single-cell RNA-Seq in further studies to better characterize cellular heterogeneity. Another limitation of this study is the use of only two timepoints (0 and 10 dpi), which could have contributed to the absence of detectable expression of key cytokines observed in other models of Apicomplexa parasite infection, such as IFNγ and IL-12 [96]. The timepoint of 10 dpi was selected due to the high parasite load and low PCV observed in infected animals in previous study [26]. Earlier timepoints may be valuable for categorizing the profile of cytokines in response to the increasing number of parasites in bovine RBCs. Bastos et al. showed the expression profiles of IL-10, TNF, CXCL10, IFN, IL-6, IL-8 and IL-4 using ELISA in *B. bovis*-infected adult animals, at various timepoints (0, 3, 6 9, and 11 dpi) [26]. However, the transcriptomic profile of these cytokines during the early stages of the acute phase of infection has not yet been described.

## Conclusions

Host immune responses to acute *B. bovis* infection remain poorly characterized, and further research is needed to elucidate the underlying mechanisms of immunity. Immunity to *Babesia* infection involves both innate and adaptative responses, and both are essential for the detection and control of blood-stage parasite replication during acute disease in a naïve host. In contrast, it is primarily antibody-mediated responses that play a key role in maintaining immunity during persistent infection. In bovine babesiosis, it is the timing, type and intensity of inflammatory cytokine responses during early infection that determines whether the disease resolves without complications, progresses to severe illness or results in death. These characteristics may be associated with host age, as older animals are generally more susceptible to fatal outcome from *B. bovis* infection.

In this RNA-Seq analysis, we identified numerous immune-related genes that were modulated in response to *Babesia* infection, including those encoding cytokines and chemokines, components of the complement system, cell signaling pathway molecules and surface receptors potentially involved in PAMP recognition, among others. However, cytokines such as IFN-γ, as well as babesiacidal molecules like NO, which are described in the literature as important for a host’s control of *B. bovis*, were not identified as modulated genes in this transcriptome at the analyzed timepoint. This may indicate that this cytokine is not produced by PBMCs, or that is produced at earlier stages by these cells. Finally, our results indicate a dual host immune response to *B. bovis* infection, with strong innate activation occurring simultaneously with regulatory mechanisms, along with complement activation and apoptosis. The response of PBMCs from adult cattle during the acute phase of the babesiosis at 10 dpi showed upregulation of the pro-inflammatory cytokines IL-1β, TNFα and IL-18. However, this occurred simultaneously with the production of IL-10, an anti-inflammatory cytokine. This mixed regulatory response profile observed in response to *B. bovis* has also been observed during infection with other Apicomplexa parasites, such as *T. gondii* and *Plasmodium* spp. [96]. This ‘Yin-Yang’ or ‘push–pull’ model promotes decreased inflammation and protective immune response, as well as increased protection from immunopathology and pathogen persistence [97]. This balance may result from the pathogen's mechanisms to evade the immune system; however, it ultimately plays crucial role in reducing tissue damage caused by inflammation following infection [97].

A large number of genes primarily associated with the non-immune system were also identified in this RNA-Seq analysis. These genes are potentially involved in cell proliferation, migration, development, energy production, protein–protein interactions, molecular transport and flagella assembly, among others.

Although this study identified the expression of numerous immune- and non-immune-related genes, the specific functions and roles of most of these genes in host recognition and control of *B. bovis*, as well as their association with the clinical manifestations of babesiosis, remain largely undefined. Furthermore, it cannot be assumed that these genes are expressed exclusively in response to *B. bovis* infection. Therefore, further applied experimental research is necessary to validate the roles of the genes identified in this study.

The results found in this transcriptome increase our understanding of the molecular interactions between *Babesia* and the host immune system and may serve as a solid basis for supporting the development of more effective immune control strategies for bovine babesiosis.

## Supplementary Information

**Additional file 1: Table S1. **Total number of transcripts, including the upregulated and downregulated differentially expressed transcripts in response to *B. bovis* infection found in this study.

**Additional file 2: Table S2. **Primers used in this study for validation of RNA-seq by RT-qPCR.

**Additional file 3: Table S3. **Count of mRNA related, and transcripts associated with non-coding RNAs.

**Additional file 4: Table S4.** Summary statistics.

**Additional file 5: Figure S1. **Heatmap of a subset of rlog normalized counts for each replicate (animal #1, animal #2 and animal #3 for 0 and 10 dpi) was selected based of the bovine non-immune response genes associated with *B. bovis* infection and plotted into a cluster map with rows z-score scaled. Only genes with LFC >1.5 and FDR < 0.05 were included in the analysis. The full names of the abbreviated genes are in Table S1.

**Additional file 6: Figure S2. **Heatmap of a subset of rlog normalized counts for each replicate (animal #1, animal #2 and animal #3 for 0 and 10 dpi) was selected based of the bovine immune response genes associated with *B. bovis* infection and plotted into a cluster map with rows z-score scaled. Only genes with LFC >1.5 and FDR < 0.05 were included in the analysis. The full names of the abbreviated genes are in Table S1.

## Data Availability

The RNA-Seq data for these samples are available in the NCBI Gene Expression Omnibus (https://www.ncbi.nlm.nih.gov/geo/), under accession number GSE299675.

## Abbreviations

DC: Dendritic cell
IFN: Interferon
IL: Interleukin
iRBC: Infected red blood cell
NK: Natural killer cell
PAMP: Pathogen-associated molecular pattern
PBMC: Peripheral blood mononuclear cell
PRR: Pathogen recognition receptor
RBC: Red blood cell
RNA-Seq: RNA sequencing
TNF: Tumor necrosis factor
