# Supplementary material for: Transcriptome profiling of peripheral blood mononuclear cells from highly susceptible adult cattle infected with a virulent strain of Babesia bovis
Source: Parasit Vectors. 2025 Dec 15;18:503. doi: 10.1186/s13071-025-07126-x (PMC12706890; doi:10.1186/s13071-025-07126-x)
Supplement: Supplementary file 2 — Additional file 2: Table S2. Primers used in this study for validation of RNA-seq by RT-qPCR. [file 13071_2025_7126_MOESM2_ESM.pdf]

Table S2. Primers used in this study for validation of RNA-seq by RT-qPCR.

Gene	Forward Sequence (5'→ 3')	Reverse Sequence (5'→ 3')	Amplicon Size (bp)	Modulation on *Babesia bovis*-infected animals by RNA-Seq
TNIP3	TGCCCTCATGAACCACAAGA	TTCAGACTTCCCCAAACGGT	124	Up
ADGRE2	TTGCCCAACTCAGAACAGCA	GTGCAGTGACTCATTGTTCAGG	102	Up
CACNA	TCCGAAAGAGGCTGGAGAAA	AGCACCAGGTCCACTTTTGT	130	Down
DAB2	TCAGGGACAACACAAGCAGA	CATCGCGGGCAATGAAAGAA	128	Down
ABCC8	TTCTTGTGCCCAAACCTCTG	TGATGACAGCCGCTCCAATT	106	Down
GAPDH	TCTGGCAAAGTGGACATCGT	TGACTGTGCCGTTGAACTTG	109	-

TNIP3: TNFAIP3 interacting protein 3; ADGRE2: adhesion G protein-coupled receptor E2; CACNA: calcium channel, voltage-dependent, alpha 2/delta subunit 1; DAB2: clathrin adaptor protein; ABCC8: ATP binding cassette subfamily C member 8; Reference gene - GAPDH: glyceraldehyde-3-phosphate dehydrogenase
