# Supplementary material for: Transcriptome profiling of peripheral blood mononuclear cells from highly susceptible adult cattle infected with a virulent strain of Babesia bovis
Source: Parasit Vectors. 2025 Dec 15;18:503. doi: 10.1186/s13071-025-07126-x (PMC12706890; doi:10.1186/s13071-025-07126-x)
Supplement: Supplementary file 3 — Additional file 3: Table S3. Count of mRNA related, and transcripts associated with non-coding RNAs. [file 13071_2025_7126_MOESM3_ESM.pdf]

Table S3: Count of mRNA related, and transcripts associated with non-coding RNAs.

Annotation	Count
mRNA-intron	56,037
mRNA-exon	8,516
lnc_RNA-intron	4,398
primary_transcript-promoter	1,699
mRNA-promoter	1,680
tRNA-promoter	1,644
lnc_RNA-exon	1,234
snRNA-promoter	1,002
snoRNA-promoter	720
mRNA-TTS	667
transcript-exon	303
lnc_RNA-TTS	238
lnc_RNA-promoter	225
transcript-intron	179
V_gene_segment-exon	101
transcript-TTS	60
primary_transcript-TTS	56
snoRNA-TTS	41
V_gene_segment-intron	36
tRNA-TTS	30
snRNA-TTS	29
transcript-promoter	28
snoRNA-exon	24
guide_RNA-promoter	23
rRNA-promoter	13
C_gene_segment-intron	11
guide_RNA-exon	5
V_gene_segment-TTS	4
C_gene_segment-exon	3
V_gene_segment-promoter	2
telomerase_RNA-exon	1
guide_RNA-TTS	1
rRNA-exon	1
C_gene_segment-TTS	1
