# Supplementary material for: Transcriptome profiling of peripheral blood mononuclear cells from highly susceptible adult cattle infected with a virulent strain of Babesia bovis
Source: Parasit Vectors. 2025 Dec 15;18:503. doi: 10.1186/s13071-025-07126-x (PMC12706890; doi:10.1186/s13071-025-07126-x)
Supplement: Supplementary file 4 — Additional file 4: Table S4. Summary statistics. [file 13071_2025_7126_MOESM4_ESM.pdf]

Table S4. Summary statistics.

Sample	Days post infection	Raw Reads	Trimmed Reads	Alignment Rate (%)	Unique Positions	Total Tags	Tags Per Position	%GC
Calf #1	0 dpi	32,521,187	32,519,922	93.45	43,986,026	29,599,283.5	0.245	0.447
Calf #2	0 dpi	26,562,561	26,561,496	94.28	36,222,332	24,283,389.0	0.239	0.443
Calf #3	0 dpi	23,749,369	23,748,739	94.63	33,904,657	21,965,578.0	0.211	0.437
Calf #1	10 dpi	43,119,482	43,118,205	93.43	52,481,694	39,222,154.5	0.327	0.445
Calf #2	10 dpi	26,611,373	26,610,685	94.71	34,947,823	24,431,013.0	0.266	0.454
Calf #3	10 dpi	32,059,811	31,945,126	94.16	42,399,024	29,485,507.5	0.27	0.443
