# Supplementary figures and images for: Transcriptome profiling of peripheral blood mononuclear cells from highly susceptible adult cattle infected with a virulent strain of Babesia bovis
Source: Parasit Vectors. 2025 Dec 15;18:503. doi: 10.1186/s13071-025-07126-x (PMC12706890; doi:10.1186/s13071-025-07126-x)

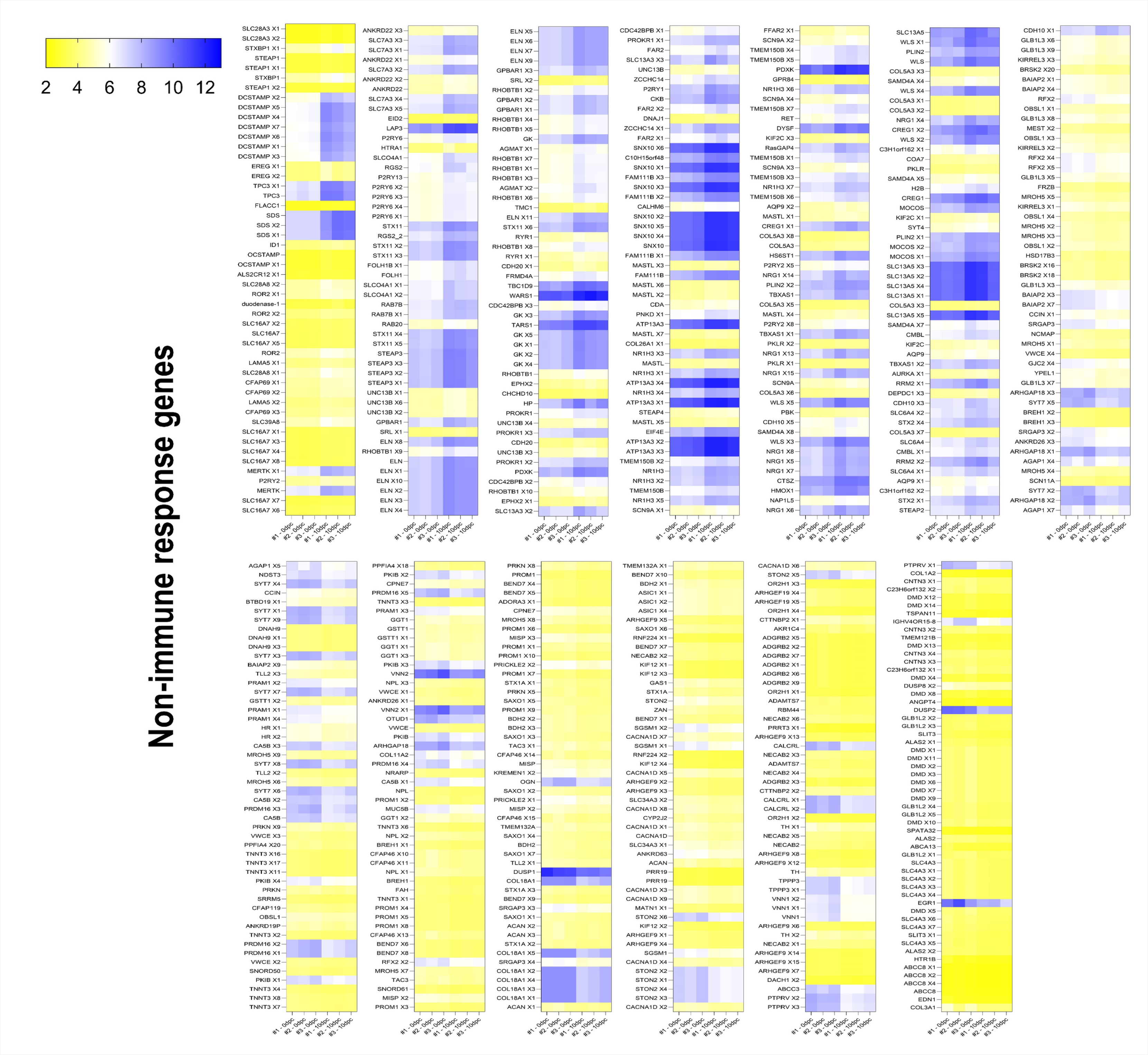

Supplement: Supplementary file 5 — Additional file 5: Figure S1. Heatmap of a subset of rlog normalized counts for each replicate (animal #1, animal #2 and animal #3 for 0 and 10 dpi) was selected based of the bovine non-immune response genes associated with B. bovis infection and plotted into a cluster map with rows z-score scaled. Only genes with LFC >1.5 and FDR < 0.05 were included in the analysis. The full names of the abbreviated genes are in Table S1. [file 13071_2025_7126_MOESM5_ESM.tif]

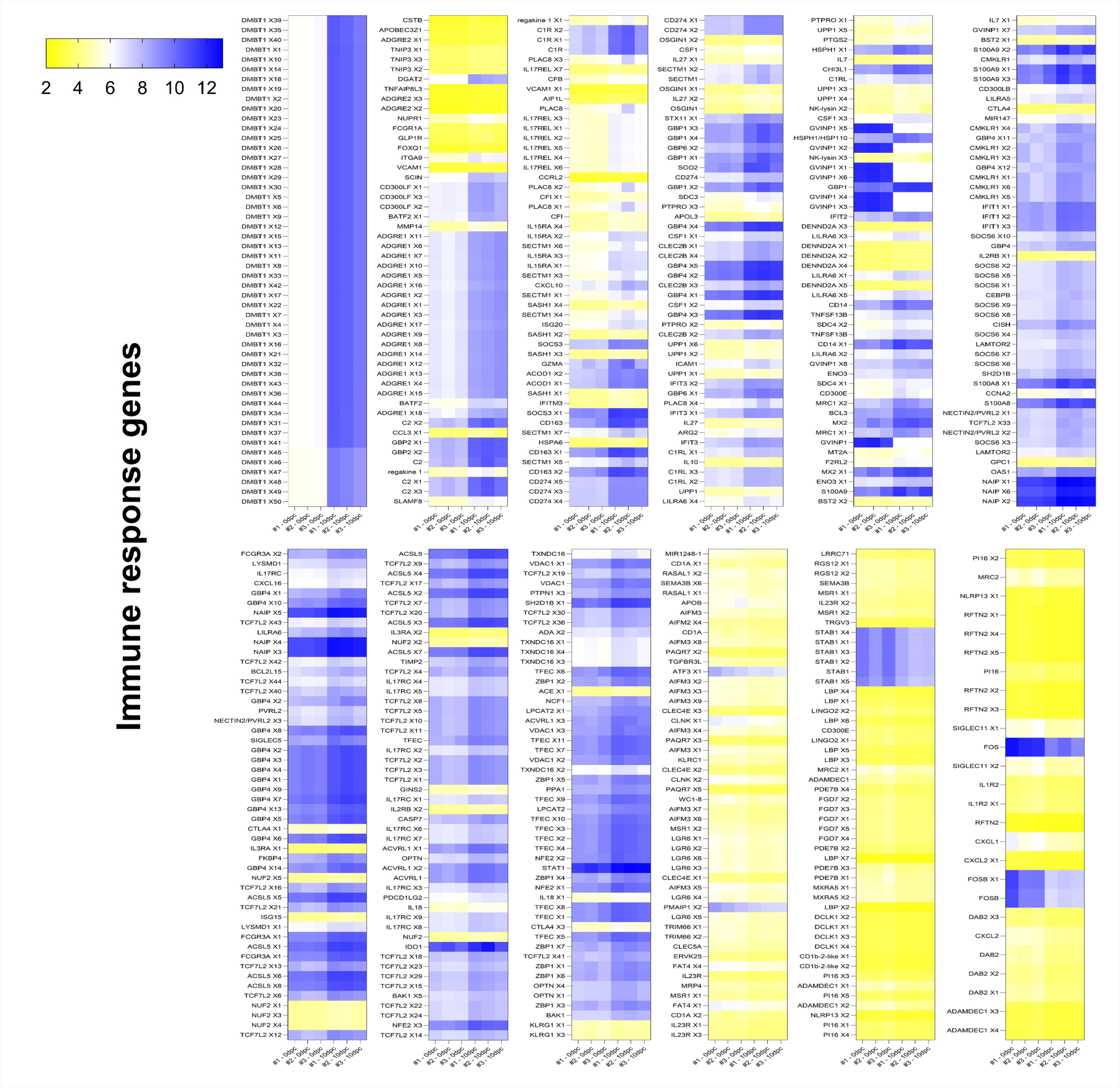

Supplement: Supplementary file 6 — Additional file 6: Figure S2. Heatmap of a subset of rlog normalized counts for each replicate (animal #1, animal #2 and animal #3 for 0 and 10 dpi) was selected based of the bovine immune response genes associated with B. bovis infection and plotted into a cluster map with rows z-score scaled. Only genes with LFC >1.5 and FDR < 0.05 were included in the analysis. The full names of the abbreviated genes are in Table S1. [file 13071_2025_7126_MOESM6_ESM.tif]
